# Assessment of Cytotoxic, Genotoxic, and Oxidative Stress of Dibutyl Phthalate on Cultured Bovine Peripheral Lymphocytes

**DOI:** 10.1155/2022/9961513

**Published:** 2022-03-24

**Authors:** Muhammad Muddassir Ali, Taha Sahar, Sehrish Firyal, Muhammad Ijaz, Khalid Abdul Majeed, Furqan Awan, Memoona Adil, Haroon Akbar, Muhammad Imran Rashid, İbrahim Hakki Ciğerci

**Affiliations:** ^1^Institute of Biochemistry and Biotechnology, University of Veterinary and Animal Sciences, Lahore 54000, Pakistan; ^2^Department of Veterinary Medicine, University of Veterinary and Animal Sciences, Lahore 54000, Pakistan; ^3^Department of Physiology, University of Veterinary and Animal Sciences, Lahore 54000, Pakistan; ^4^Department of Epidemiology and Public Health, University of Veterinary and Animal Sciences, Lahore 54000, Pakistan; ^5^Department of Parasitology, University of Veterinary and Animal Sciences, Lahore 54000, Pakistan; ^6^Department of Molecular Biology and Genetics, Afyon Kocatepe University, Afyonkarahisar, Turkey

## Abstract

Recently, there have been numerous reports showing that phthalates have negative human health impacts and may cause several diseases such as asthma, breast cancer, obesity, type II diabetes, and male infertility. Animals are also exposed to phthalate**s** through the environment and can cause adverse health effects on them. Several studies have been found on the cytogenetic effects of dibutyl phthalate (DBP) on different organisms but no documented evidence has been found on the cytotoxic and genotoxic effects of dibutyl phthalate (DBP) on bovine cultured lymphocytes. MTT assay was performed on different series of DBP concentrations (10 *μ*M, 20 *μ*M, 30 *μ*M, 50 *μ*M, 70 *μ*M, 100 *μ*M). A concentration-dependent decrease in cell viability was observed by the DBP. The LD50, LD50/2, and 2∗LD50 were found to be 50 *μ*M, 30 *μ*M, and 80 *μ*M on bovine lymphocytes, respectively. Then, these concentrations of DBP were utilized to perform comet, micronucleus assays, and oxidative stress. A concentration-dependent increase in DNA damage, oxidative stress, and micronuclei formation was observed in lymphocytes by the DBP as compared to the control group. Highest genotoxic effects were observed at a concentration of 2∗LD50. Similarly, total oxidative stress was found higher, and antioxidative stress was lower in concentration-dependent manner by the DBP. The current study revealed a significant cytotoxic, genotoxic, and oxidative stress of DBP on cultured bovine lymphocytes.

## 1. Introduction

Phthalates are the diesters of orthophthalic acid (1, 2-benzene carboxylic acid) and commonly known as diester compounds. These lipid-containing organic compounds are produced annually in huge volumes. One million ten of phthalate is produced and used in European countries as itis used in the formation of a wide variety of materials [1]. However, their biological negative impacts have also been associated with the many problems for the cell. Production of different reactive oxygen species (ROS) and oxidative stress is a major problem caused by many of the industrially manufactured chemicals. This may lead to genotoxicity, causing inflammatory diseases and cancer. Phthalates are conservational compounds and are recognized as endocrine disruptors and peroxisome proliferators (PPs) [2]. Phthalates are involved in carcinogenesis and pulmonary disorders [3]. *In vivo* and epidemiological studies associated structural impairment of the lung with phthalate exposure [4]. DBP is highly accessible upon inhalation. DBP considered crucial in studies of respiratory toxicity [5]. DBP is also present in exercise balls, hoses, rubber sheets, and in children's toys, including those intended for children aged 0-6 years. The characteristic concentration of DBP in an unspecified set of products for children was identified as 0.5%. The low content of DBP in children's toys and childcare articles coincides with its physicochemical characteristics (i.e., high volatility) that make DBP unsuitable for use as the primary plasticizer for PVC [6].

Phthalates are also used in the manufacturing of plastic containers, water bottles, medicine jars, infant formula milk, and nutritional supplements. Phthalate exposures from indoor sources are vapours produced from materials used for the construction of buildings, domestic fragrances, and home stuff. Different kinds of medical devices for therapeutic maintenance such as tubes for circulatory fluids, blood bags, and dialysis tubing materials are also made up of plastic which makes them softer with different kinds of PVC and organic compounds. Skin absorption can occur through direct contact with cosmetics and clothing products containing phthalate [7]. In silage, DBP is almost surely contaminated due to immigration from interaction materials such as sticking films, sails collected from rivers used during manufacture, or during farm storage [8]. So, bovines could be directly or indirectly affected by the DBP. Cytogenotoxic and oxidative effects of DBP have been already been investigated in different model organisms [9, 10] and cell lines [11–13]. But to the best of author's knowledge, no documented evidence has been observed to observe the cytogenetic and oxidative stress effects of DBP on bovine lymphocytes. As, bovines could be exposed to DBP by various ways as earlier mentioned. So, it is inevitable to explore the cytotoxic, genotoxic, and oxidative stress on bovines. Current study was designed to evaluate the cytotoxic, genotoxic effects, and oxidative stress of DBP on bovine cultured lymphocytes through MTT, comet assay, micronucleus test, total oxidant status (TOS), and total antioxidant status (TAS).

## 2. Materials and Methods

### 2.1. Chemicals and Reagents

All chemicals were of Sigma-Aldrich, Munich, Germany. Main chemicals were dibutyl phthalate (CAS No. 84074-2), low melting point agarose (A4018), normal melting point agarose (A9539), Triton X-100, basic fuchsin, ethidium bromide (EtBr), sodium hydroxide, hydrochloric acid, RMPI-1640 media, HEPES, methyltetrazolium (MTT), EDTA, etc. All chemicals were of analytical grade.

### 2.2. Culturing and Growth of Bovine Lymphocytes

A total of 6 healthy cattle were selected, and their blood samples were taken in sterilized EDTA syringes and shifted to clean falcon tubes. Lymphocytes were isolated using histopaque-1077 technique (Sigma-Aldrich, Munich, Germany). Isolated lymphocytes were observed under the microscope for analyzing viable cells. Cells were cultured by using the RMPI-1640 culture medium having 10% FBS, 1% penicillin-streptomycin, amphotericin-B, and 1% HEPES. Cells were cultured using this growth medium in culture flasks and incubated with the appropriate conditions of 5% CO_2_, 2% O_2_, and 95% N_2_ at 37°C in incubator.

### 2.3. MTT Assay

The MTT assay was performed for the cytotoxic assessment after 24 h incubation in 96 well plates. Total of 1 × 10^5^ cells were cultured in each well for 24 h. After 24 h, viable cells were exposed to the different concentrations of DBP from 10 *μ*M, 20 *μ*M, 30 *μ*M, 50 *μ*M, 70 *μ*M, and 100 *μ*M in triplicate for 24 h. 0.1% dimethyl sulfoxide (DMSO) was taken as solvent control, whereas distilled water was taken as a negative control. After 24 h, MTT reagent (10 *μ*L) was added in all wells including controls and again incubated for 3 h. After 3 h of incubation, the medium was removed, and 100 *μ*L DMSO was added, gently shaken, and kept at room temperature for 30 min. Absorbance was measured at 630 nm, and average values were calculated for all concentrations and controls [14].

### 2.4. TAS and TOS Measurement

TOS and TAS levels were determine by Rel Assay Diagnostic kit RL0024 and RL0017 (Total Oxidant Kit, 3. Generation Antioxidant Assay Kit, Mega Tıp Korea, Gyeonggi-do, Korea). Procedure was adopted as per manufacturer protocol. Sample oxidants oxidized the ferrous ion-o-dianisidine complex into ferric ion. Colored complex was formed by ferric ion in the presence of acidic medium. Spectrophotometer was used to determine the total amount of oxidant molecules present in the sample. TOS was analyzed spectrophotometrically at 530 nm while TAS was analyzed spectrophotometrically at 660 nm. TOS and TAS were calculated as reported by Ali and his colleagues [14].

### 2.5. Alkaline Comet Assay

Comet assay was performed as described earlier by Liman et al. [15]. Briefly, the assay was performed with LD50, LD50/2, and 2∗LD50 of dibutyl phthalate at which 25%, 50%, and 75% of cells remained viable with MTT. The doses for LD50 , LD50/2 and 2∗LD50 were found as 50µM, 30µ M, and 80µM respectively. ∗The doses were treated with cells (1.5 × 10^8^) in 25 cm^2^ flasks and incubated for 24. Briefly, the treated cells (10 *μ*L) were mixed with low melting agarose (1.5%) and loaded in 1% normal melting agarose precoated slides. Slides were kept on ice for 5 min followed by lysis in lysis buffer (2.5 M NaCl, 100 mM Trisma Base, 100 Mm EDTA, 1% Triton X-100, and 10% DMSO, pH = 10) at 4°C. Lysis was performed for an hour, and then, slides were kept in electrophoresis buffer solution (1 mM EDTA, 300 mM NaOH, pH > 13) for 20 min. Electrophoresis was further carried out at 4°C for 20 min at 25 mV and 300 Amp. Slides were stained with EtBr (20 *μ*g/mL) to observe under fluorescence microscope at 40x. Level of DNA damage was observed for each concentration and control group and expressed as an arbitrary unit (AU). A total of 50 nuclei were randomly counted from each slide per each concentration and scored from 0 to 4 depending on the level of DNA damage (0 = no damage, 1 = mild damage, 2 = moderate, 3 = severe, 4 = complete DNA damage). Three slides were made from each experiment [16, 17].

### 2.6. Micronucleus Test

Micronucleus test was carried out as described by the Cigerci and his colleagues [18]. Micronucleus test was carried out by employing LD50, LD50/2, 2∗LD50 concentrations of DBP on cultured lymphocytes. The cells (1.5 × 10^8^) were treated with these concentrations in 25 cm^2^ flasks and incubated for 24 h. Treated cells were centrifuged with 1% KCL. Then, pellets were centrifuged with fixative 1 (50 mL fixative II and 50 mL 0.09%NaCl) and fixative 11 (40 mL glacial acetic acid and 200 mL methanol), respectively. Samples were coated on prechilled wet slides, air-dried, and stained with 10% Giemsa stain. Then, slides were washed with distilled water and observed under a compound microscope for the identification of micronuclei and binuclei. The total of 500 cells was counted from each slide. All experiments were repeated in triplicate.

### 2.7. Statistical Analysis

One-way ANOVA was applied to analyze the results of an alkaline comet and MN test using SPSS version 15.0 for the Windows software. Duncan test was applied in the SPSS package to compare different concentration of groups. Statistical significant value *p* < 0.05 was kept to represent significance difference.

## 3. Results

**Figure 1:**
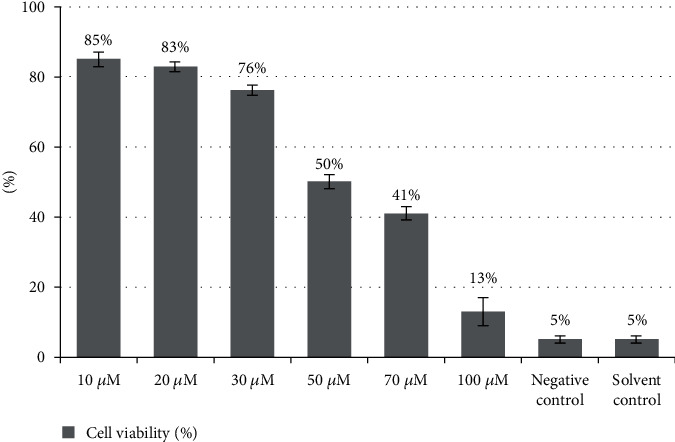
Percentage of viable bovine peripheral lymphocytes treated with different concentrations of DBP for 24 h. Series of concentrations were employed on bovine peripheral lymphocytes and shown in *y*-axis whereas % viability is shown on *y*-axis.

The effect of DBP concentrations on cell viability using MTT assay is shown in Figure 1. DBP induced cell death in a concentration-dependent way. The effective concentration of DBP causing 50% cell death (LD50) was 50 *μ*M after 24 h of exposure to DBP concentration. The LD50, LD50/2, and 2∗LD50 were found as 50 *μ*M, 30 *μ*M, and 80 *μ*M on bovine lymphocytes, respectively.

**Figure 2:**
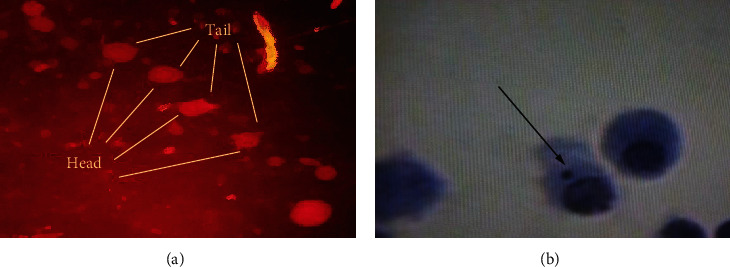
Microscopic view of (a) comet cells along with head and tail and (b) normal cells along with micronuclei formation due to DBP exposure. Direction of arrow is showing the micronuclei in (b).

**Figure 3:**
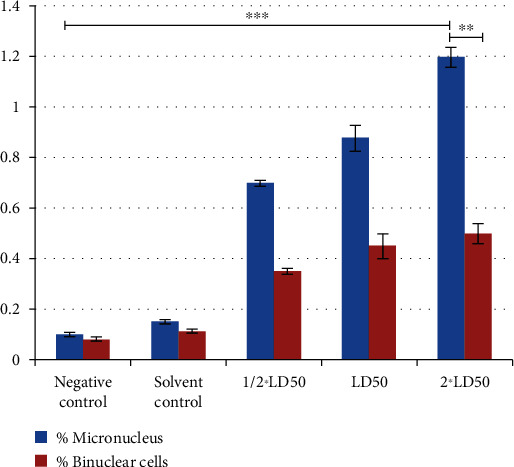
Number of micronuclei and binuclei after exposure of DBP on bovine lymphocytes. Significant difference exists between 2∗LD50 and negative control whereas 2∗LD50 of micronucleus is also significantly different from 2∗LD50 of binuclear cells. ^∗∗^*p* value < 0.01; ^∗∗∗^*p* value < 0.001.

Total DNA damage score induced by the DBP is represented in Table 1. Highest DNA damage (Figure 2(a)) was observed at the 2∗LD50, and the least was observed by the LD50/2 of DBP in bovine lymphocytes compared to the control group. There was a statistical difference among the group (*p* < 0.05). Similarly, a significant effect of induction of micronuclei and binuclei has been observed at all the three concentrations of DBP (Figure 2(b)) compared to the negative control group as shown in Figure 3. A more number of micronuclei were observed compared to the binuclei.

TOS was increased in concentration-dependent manner whereas a decrease in TAS values was observed with increase in concentrations of DBP. TOS values were significantly higher in different treatment groups of DBP as compared to TAS values (Table 2).

## 4. Discussion

Low molecular weight phthalates like DBP can interact with animals directly through inhalation or indirectly through the contact with plastic utensils [19]. Several carcinogenic and mutagenic effects of phthalates are being studied both in in vivo and in vitro experiments [2]. Consequently, MTT assay was used to evaluate the cytotoxic effects of DBP concentrations on bovine lymphocytes which showed a significant decrease in cell viability by DBP. Cytotoxic effects of phthalates and their alternatives have been observed already on different cell lines like L929 and kidney cells [20, 21]. They conceived that cell death could be attributed due to arrest of cell cycle or depletion in mitochondrial energy that could lead to decreased cell viability with a higher concentration of plasticizer [22].

Similarly, genotoxic effects were also observed by the DBP on bovine lymphocytes. Previously, different types of phthalates have already shown genotoxicity [23–26] in different assay systems. Comet assay and MN were used to evaluate the cytogenetic effects due to their different way of exploring genotoxic effects. The comet assay can detect DNA damage, i.e., single-strand breakage or other aberrations, such as alkali–labile sites, DNA cross-links [27, 28], and incomplete excision repair events [29]. It offers extensive benefits over other cytogenetic methods like chromosome aberrations, sister chromatid exchanges as the cells need not be mitotically active for comet assay [30]. Therefore, it has been broadly used in the areas of genetic toxicology and environmental biomonitoring [28]. Whereas in MN, micronuclei generation results due to the fragmentation of chromatids or lagging acentric chromosomes due to disrepair of DNA breaks [31].

Higher DNA damage and formation of micronuclei in the current study were suggestive of adverse genotoxic effects due to DBP on bovine lymphocytes. Current results are in agreement with the findings of Kleinsasser and his colleagues [11]who demonstrated the DNA damage caused by DBP in human lymphocytes and used the single-cell electrophoresis technique (comet assay) for identification of damage. Highest estrogenic activity studied by investigating the induced proliferation of human breast cancer cell MCF-7/BUS by a low dose of DBP even at 16 *μ*M and 35 *μ*M [32]. DBP also suppressed the cell growth by inducing apoptosis in leukaemia cells [33].

Genotoxic effects exerted by phthalates and their metabolites were observed at chromosome and DNA level [2]. These genotoxic effects might be the result of increased generation of oxidative stress [34]. Phthalate-induced oxidative stress plays vital role in the progress of lung diseases. It has been revealed by many studies on different human cell types that DBP induce genotoxic lesions resulting stress [35]. DNA base lesion was observed in a study conducted on peripheral blood mononuclear cells (human) to reveal genotoxic effects after increasing DBP treatment. Single and double DNA strand breaks induced by DBP; these DNA damages were more adverse as compared to its metabolite monobutyl phthalate [34].

Genotoxic effects of phthalates have also been observed at the higher concentrations by other studies [36–38]. Genotoxic effects at higher concentrations might be due to indirect genotoxic effects subsequently from primary toxicity, impurities, or limited solubility, whereas no mutagenic effects were observed by the phthalates by using the bacterial strains of Salmonella typhimurium [39], and no genotoxicity was observed by the phthalate esters even at a higher level [24]. Phthalate and their alternatives even induced DNA methylation [40, 41]. Dibutyl phthalate oxidative stress and genotoxicity were studied in HepG 2 cells. It has been revealed that excessive oxidative stress in HepG 2 cells leads to cell death [13]. DBP also caused the increase in oxidative stress and inflammation resulted in grass carp hepatocytes and rise of apoptosis-related markers and protein levels.DBP is involved in the increase of apoptotic cells and it was also confirmed by staining (AO/EB and Hoechst) and flow cytometry [42]. These controversies in findings in various studies could be due to different assay systems, cell types, or variations in experimental conditions.

## 5. Conclusion

Different studies have been observed by the DBP induced toxic effects on different model organisms. However, no documented evidence has been found on the genotoxic effects of DBP on bovine peripheral lymphocytes. Findings of the current study provide an insight into potential cytotoxic, genotoxic, and oxidative stress effects of DBP on bovine lymphocytes. It can be predicted that accidental exposure of animals to DBP is hazardous at their cellular and genetic level. In future, this study would provide a potential information regarding the cautious use of DBP in products of animal use.

## Figures and Tables

**Table 1:** DNA damage score (expressed in arbitrary unit) after DBP exposure on bovine lymphocytes. Different symbols are representing the statistical difference among different exposed groups (*p* < 0.05).

Groups	Mean ± S.D (arbitrary unit)
Negative control	3 ± 1.4^a^
Solvent control	5 + 1.2^a^
30 *μ*M	39 ± 3.1^b^
50 *μ*M	60 ± 2.1^c^
80 *μ*M	70 ± 1.7^d^

**Table 2:** Total oxidative status (TOS) and total antioxidative status (TAS) after DBP exposure on bovine lymphocytes.

Groups	TOS (*μ*mol H_2_O_2_ equivalent/L) ± SD	TAS (mmol Trolox equivalent/L) ± SD
Negative control	0.91 ± 1.6	4.20 ± 3.04
Solvent control	1.91 ± 1.4	3.22 ± 2.03
30 *μ*M	4.95 ± 1.02	4.12 ± 2.2
50 *μ*M	6.91 ± 2.4	3.11 ± 1.09
80 *μ*M	8.17 ± 1.1	2.22 ± 2.08

## Data Availability

All the data is included in the article with transparency, and additional raw data can be provided on demand.
